# A disease-specific iPS cell resource for studying rare and intractable diseases

**DOI:** 10.1186/s41232-023-00294-2

**Published:** 2023-09-08

**Authors:** Megumu K. Saito, Mitsujiro Osawa, Nao Tsuchida, Kotaro Shiraishi, Akira Niwa, Knut Woltjen, Isao Asaka, Katsuhisa Ogata, Suminobu Ito, Shuzo Kobayashi, Shinya Yamanaka

**Affiliations:** 1 Department of Clinical Application, Center for iPS Cell Research and Application, Kyoto University, Kyoto, 6068507 Japan; 2 Clinical Research Center, National Hospital Organization Headquarters, Tokyo, 1528621 Japan; 3 Information Security Office, Center for iPS Cell Research and Application, Kyoto University, Kyoto, 6068507 Japan; 4 Department of Life Science Frontiers, Center for iPS Cell Research and Application, Kyoto University, Kyoto, 6068507 Japan; 5 Department of Fundamental Cell Technology, Center for iPS Cell Research and Application, Kyoto University, Kyoto, 6068507 Japan; 6 National Hospital Organization Higashisaitama National Hospital, Hasuda, 3490196 Japan; 7 Kidney Disease and Transplant Center, Shonan Kamakura General Hospital, Kamakura, 2478533 Japan; 8 CiRA Foundation, Kyoto, 6068397 Japan; 9 Gladstone Institute of Cardiovascular Disease, San Francisco, CA 94158 USA

**Keywords:** iPS cells, Designated diseases, Rare and intractable diseases, Reprogramming

## Abstract

**Background:**

Disease-specific induced pluripotent stem cells (iPSCs) are useful tools for pathological analysis and diagnosis of rare diseases. Given the limited available resources, banking such disease-derived iPSCs and promoting their widespread use would be a promising approach for untangling the mysteries of rare diseases. Herein, we comprehensively established iPSCs from patients with designated intractable diseases in Japan and evaluated their properties to enrich rare disease iPSC resources.

**Methods:**

Patients with designated intractable diseases were recruited for the study and blood samples were collected after written informed consent was obtained from the patients or their guardians. From the obtained samples, iPSCs were established using the episomal method. The established iPSCs were deposited in a cell bank.

**Results:**

We established 1,532 iPSC clones from 259 patients with 139 designated intractable diseases. The efficiency of iPSC establishment did not vary based on age and sex. Most iPSC clones originated from non-T and non-B hematopoietic cells. All iPSC clones expressed key transcription factors, *OCT3/4* (range 0.27–1.51; mean 0.79) and *NANOG* (range 0.15–3.03; mean 1.00), relative to the reference 201B7 iPSC clone.

**Conclusions:**

These newly established iPSCs are readily available to the researchers and can prove to be a useful resource for research on rare intractable diseases.

**Supplementary Information:**

The online version contains supplementary material available at 10.1186/s41232-023-00294-2.

## Introduction

Rare diseases are defined as those that affect a relatively small number of people in the population. According to the definition of the European Union (EU), rare diseases are those that affect less than one person per 2,000 people (0.05% of the EU population), and in the United States (US), they are known to affect less than 200,000 people (about 0.06% of the US population) [1] The total number of rare diseases is estimated to be more than 7,000 [1]. For such rare diseases, patients usually cannot receive proper diagnosis and consensus therapeutic strategies have not been established. Global consortia and databases have been established to overcome these issues and establish diagnostic and therapeutic approaches [2, 3].

One of the challenges in the research and treatment of rare diseases is that the small number of patients makes it difficult to design studies for the development of diagnostic and therapeutic approaches; specifically, large-scale controlled trials are often difficult to conduct. In addition, access to patient samples is often limited. To overcome these problems, induced pluripotent stem cell (iPSC)-based disease modeling is a promising approach [4, 5]. iPSCs can be easily established from patient's somatic cells such as peripheral blood mononuclear cells (PBMCs) [6, 7], fibroblasts [8, 9], and urine [10]. Since iPSCs inherit the genetic information of their donor, they are particularly useful for the analysis of genetic diseases, which are estimated to account for approximately 80% of rare diseases [11]. Recent advances in differentiation technology have enabled the differentiation of iPSCs into a wide variety of cell types [12]. In addition, the development of 3D organoid methods has enabled the modeling of complex structures of human organs, such as the brain, liver, and kidney [13–15]. Indeed, in several rare diseases, structural abnormalities of the cerebrum have been reproduced using iPSC-derived cerebral organoids [16, 17].

However, the treatment of patients with rare and intractable diseases is often time-consuming, placing a heavy mental, physical, and financial burden on the patients and their families. Therefore, in Japan, a system of designated intractable diseases was introduced in 2015 to reduce the economic burden of patients with rare intractable diseases [18]. The criteria for classifying a disease as a designated intractable disease are as follows: (1) rarity (less than 0.1% of the Japanese population), (2) unknown etiology, (3) no effective treatment, (4) need for long-term treatment, and (5) existence of objective diagnostic criteria. Patients with designated intractable diseases are eligible to receive subsidies for medical expenses in exchange for providing medical information in a format designated for each disease to the Ministry of Health, Labor, and Welfare, Japan. As of December 2021, 338 diseases had been classified as designated intractable diseases. This system also serves as a comprehensive database, covering more than 90% of all newly designated intractable disease cases in Japan [18]. It also serves as a traceable database because medical information is continuously collected after registration.

Because iPSCs derived from patients with designated intractable diseases are accompanied by reliable medical history information, they can prove to be a useful resource for the analysis of rare diseases. Therefore, in this study, we established iPSCs from patients with designated intractable diseases in Japan as a resource to promote the research pertaining to the rare diseases. We recruited 259 patients with 139 designated diseases and established 1,532 clones on a unified platform. All iPSCs have been deposited at the RIKEN BioResource Center, where they would be available for academic use; notably, although these iPSCs are not available for direct selling, they can still be used for commercial research. We believe that these iPSCs can be effectively utilized as a resource for the construction of pathological models for a wide range of rare diseases.

## Methods

### Study approval

This study was approved by the Ethics Committees of Kyoto University (G687), Shonan Kamakura General Hospital, and National Hospital Organization (NHO) (H26-NHO (shitei)-05) in Japan. Written informed consent was obtained from all patients or their guardians in accordance with the Declaration of Helsinki.

### Donor recruitment and sample collection

At the Shonan Kamakura General Hospital, electronic medical records were searched for patients with designated intractable diseases, and medical coordinators and doctors obtained written informed consent from the candidates. In the NHO, each hospital belonging to the organization recruited patients with target diseases and sent their medical information to the headquarter. The headquarter scrutinized the medical information and selected eligible patients only if they met the criteria for the target disease. Subsequently, physicians from each hospital obtained written informed consent and collected the blood samples. Anonymization was performed at each facility. Peripheral blood mononuclear cells were isolated from the donor blood and cryopreserved. All donors underwent blood tests to exclude the presence of infection with hepatitis B, hepatitis C, human T-cell leukemia type 1, and human immunodeficiency viruses.

### Establishment and maintenance of iPSCs

iPSCs were established from blood cells using previously described methods with marginal modifications [6]. Briefly, frozen peripheral blood mononuclear cells were thawed and cultured in StemSpan-ACF medium (STEMCELL Technologies, Vancouver, British Columbia, Canada) supplemented with stem cell factor (SCF; 100 ng/mL), thyroperoxidase (TPO; 100 ng/mL), Flt3 ligand (100 ng/mL), IL-6 (50 ng/mL), and IL-3 (10 ng/mL) for five days. SCF, TPO, Flt3 ligand, IL-6, and IL-3 were purchased from R&D Systems (Minneapolis, MN, USA). The episomal plasmids pCE-hOCT3/4, pCE-hSK, pCE-hUL, pCE-mp53DD, and pCXBEBNA1 were then co-transfected into 2 × 10^6^ cultured PBMCs in a Nucleofector 2b device, using the Human CD34 cell Nucleofector kit (Lonza, Basel, Switzerland). The cells were dispensed into two-fold serial dilutions (six times) and plated into six wells of 6-well plates coated with Laminin511 E8 fragment matrix (iMatrix-511, Nippi, Tokyo, Japan) containing 1.5 mL of StemSpan-ACF medium containing the above-mentioned cytokines. Subsequently, 1.5 mL of complete StemFit AK03 medium was added every two days for three times, and the culture medium was replaced with 1.5 mL of complete StemFit AK03 medium at day 8 after the transduction. The colonies were picked up 2–3 weeks after the transduction and plated into 12-well plates coated with iMatrix-511 and cultured in 0.8 mL of complete StemFit AK03 medium containing Y27362 (10 μM, Wako, Osaka, Japan) for one day; the medium was then replaced with 0.8 mL of complete StemFit AK03 medium without Y27362. Five to seven days later, the cells were passaged again. Six clones were selected to generate passage two (P2) stocks. iPSCs were cultured on iMatrix-511-coated tissue culture plates using StemFit AK03 medium at 37 °C in an atmosphere containing 5% CO_2_ and 21% O_2_. Cells were passaged by dissociating them using TrypLE Select (Life Technologies, Gaithersburg, MD, USA). All clones were confirmed to be free of mycoplasma contamination prior to their deposition in the cell bank.

### Microscopy

Morphological images were captured using an Olympus CKX41 microscope with a PlanApo 10 × /0.75 objective lens (Olympus, Tokyo, Japan) and a Nikon digital camera DS-Fil.

### DNA and RNA extraction and cDNA synthesis

Genomic DNA and total RNA were extracted using the AllPrep DNA/RNA Mini Kit (Qiagen), following the manufacturer’s instructions. Genomic DNA was diluted to 25 ng/mL using distilled water. cDNA was synthesized using PrimeScript™ RT Master Mix (TaKaRa) from 500 ng of total RNA and diluted 1:10 in RNase-free water for *OCT3/4* and *NANOG* mRNA expression analysis.

### OCT3/4 and NANOG mRNA expression analysis

The mRNA expression of pluripotent stem cell markers, *OCT3/4* and *NANOG*, was confirmed by quantitative real-time PCR (qRT-PCR) with TaqMan™ assay using StepOnePlus™ Real-Time PCR System (Thermo Fisher). Primer and probe sequences are listed in Supplementary Table S1. The expression values of the target genes were processed using the ΔΔCt method by normalization to *GAPDH* expression from the same cDNA templates, and the average of relative quantities (RQ) in comparison to the control 201B7 line are shown.

### Residual plasmid analysis

The residual plasmids used for establishing the iPSCs were analyzed by TaqMan quantitative PCR using a StepOnePlus™ Real-Time PCR System (Thermo Fisher). Primer and probe sequences of CMV and EBNA1 were designed based on CAG-promoter region and the cording region of *EBNA1* gene (Supplementary Table S1). The residual plasmid numbers were determined by a standard curve method with pCE-OCT3/4 episomal plasmid of known quantity using 50 ng genomic DNA of established iPSC at passages 4 to 6.

### Cell type of origin analysis

The cell type of origin, including T cell, B cell, or non-T/non-B cell lineage, on established iPSC was analyzed by TaqMan™ quantitative PCR using StepOnePlus™ Real-Time PCR System (Thermo Fisher). The primer and probe sequences of T cell receptor (TCR) and joining region of immunoglobulin heavy chain (JH) were designed based on the sequences of TRD, T cell receptor delta locus, and IGH, immunoglobulin heavy chain locus, respectively (Supplementary Table S1). Quantitative PCR was performed using TRD, IGH, and RNaseP1 primers and probes using 20 ng genomic DNA of established iPSC at passages 4 to 6, and the number of TRD and IGH loci was analyzed using CopyCaller™ Software (Thermo Fisher) with the number of RNaseP1 loci as an internal standard. The cell type of origin was determined as T cell lineage if the number of TRD loci was 1 or 0, as B cell lineage if the number of IGH loci was 1 or 0, and as non-T/non-B cell lineage otherwise.

### Statistics

Statistical analysis was performed using Prism version 9.0 (GraphPad, San Diego, CA, USA). Each analysis method has been described in the figure legends. Statistical significance was set at *p* < 0.05.

## Results

### Donor recruitment system and donor details

**Fig. 1:**
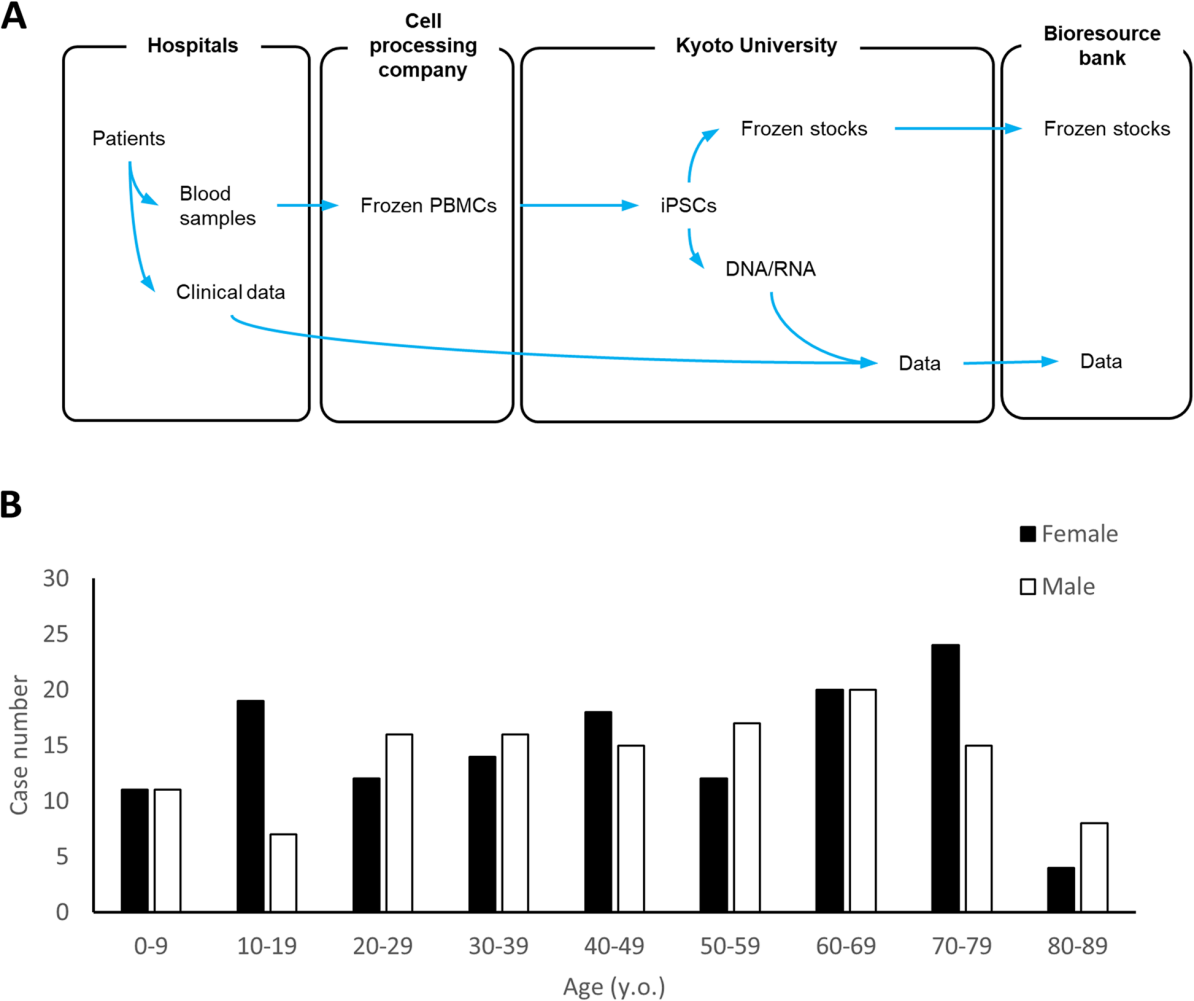
Characteristics of iPSC donors. See also Supplementary Table S2. **A** Workflow from donor recruitment to depositing iPSCs in Riken Bioresource Bank. **B** Age distribution of recruited donors

In this study, we established a pipeline from donor recruitment through iPSC establishment to iPSC deposition in a cell bank (Fig. 1A). At the start of donor recruitment, 306 intractable diseases were designated by the Japanese Ministry of Health, Labour, and Welfare. Donors with these diseases were targeted for recruitment as they had an annual medical summary required for aid. Medical institutions that participated in disease recruitment selected patients with the target diseases from electronic medical records to determine potential donors. As a result, 259 donors with 139 designated intractable diseases were recruited, and a total of 1,532 iPSC clones were established. Donor eligibility was confirmed on the basis of the diagnostic criteria for each disease.

The average age of the donors was 45.53 years. The age distribution of the patients is shown in Fig. 1B. There were 125 males and 134 females, with a sex ratio of 0.93. Designated intractable diseases were originally classified according to the International Classification of Diseases (ICD)-10. The diseases and cases from which iPSCs were established in this study were classified according to ICD-10 (Table 1). Various disease categories have been established, but the most commonly observed category is “congenital malformations, deformations, and chromosomal abnormalities” (major category XVII). Notably, the definition of designated intractable diseases has a medical administrative aspect and differs from the ICD-10 disease classification. For example, lysosomal diseases are comprehensively considered as designated intractable diseases, but can be divided into more than 30 individual disease units. Detailed donor information, including sex, age range, diagnosis, and ICD-10 classification, is presented in Supplementary Table S2.

**Table 1:** Number of donors grouped by disease category based on ICD-10

ICD-10 Chapter	Case number
I Certain infectious and parasitic diseases	3
II Neoplasms	6
III Diseases of the blood and blood-forming organs and certain disorders involving the immune mechanism	16
IV Endocrine, nutritional and metabolic diseases	44
V Mental and behavioural disorders	1
VI Diseases of the nervous system	54
IX Diseases of the circulatory system	15
X Diseases of the respiratory system	5
XI Diseases of the digestive system	18
XII Diseases of the skin and subcutaneous tissue	10
XIII Diseases of the musculoskeletal system and connective tissue	39
XIV Diseases of the genitourinary system	8
XVII Congenital malformations, deformations and chromosomal abnormalities	40
Total	259

### Reprogramming

**Fig. 2:**
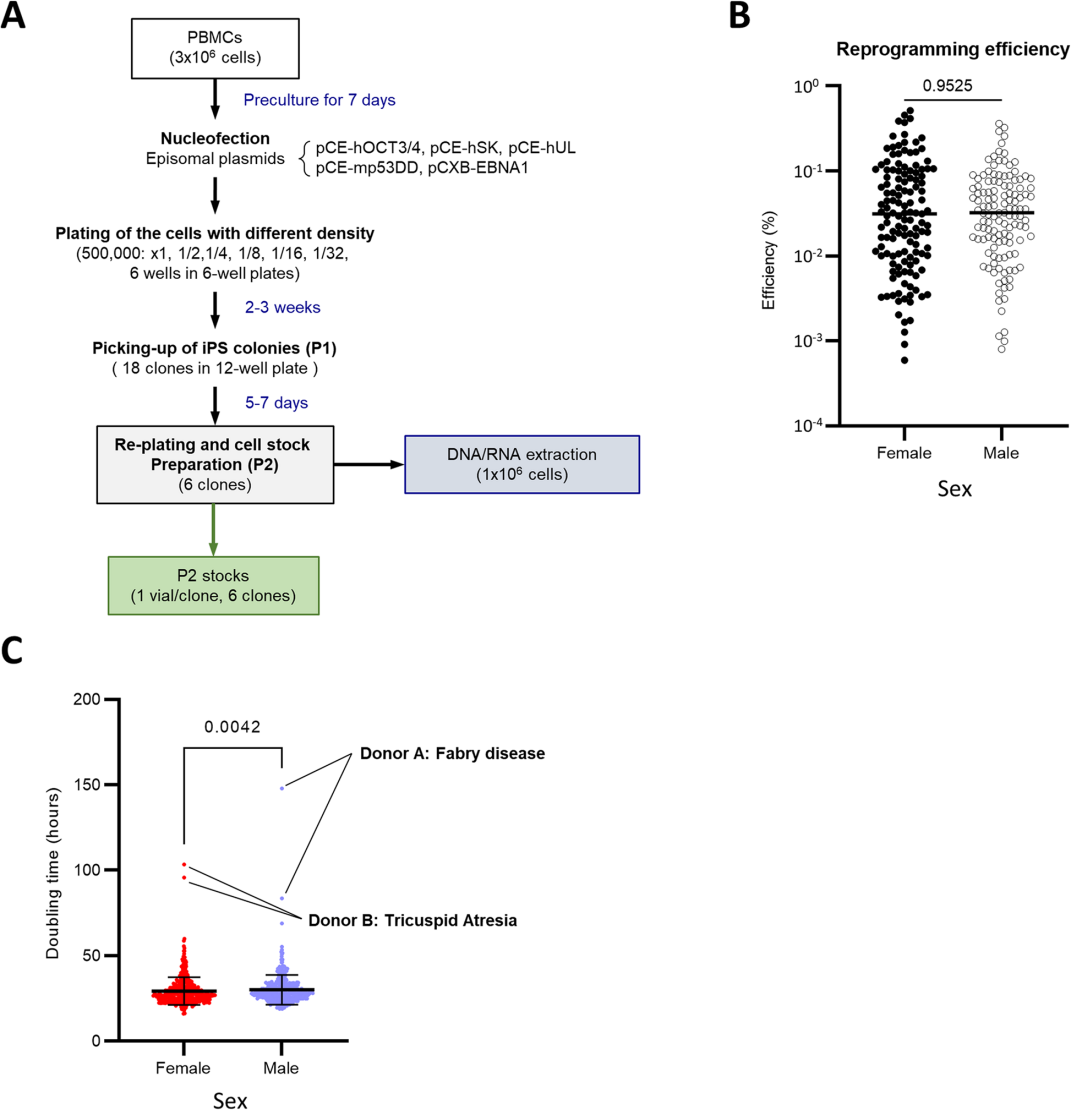
Establishment of iPSC lines. See also Supplementary Fig. 1. **A** Flowchart depicting iPSC establishment. **B** Relationship between sex and reprogramming efficiency (%). **C** Relationship between sex and doubling time of established iPSC lines. (total *n* = 1,039; female *n* = 535; male *n* = 504). **B**, **C** Statistical analysis was performed using Mann–Whitney U test

Frozen peripheral blood mononuclear cells (PBMCs) were thawed and reprogrammed by introducing episomal vectors encoding *OCT3/4, SOX2, KLF4, LIN28, LMYC*, and p53 dominant-negative fragments (Fig. 2A). In all recruited cases, iPSC clones were successfully established and no cases of reprogramming failure were recorded. For 13 donors, only five or fewer iPSC lines could be deposited, but for the remaining 246 donors (95.0%), six iPSC lines were successfully established and deposited. The average reprogramming efficiency was 0.06%, and there was no significant difference in reprogramming efficiency according to sex (Fig. 2B). No correlation between age and reprogramming efficiency was observed partly because of the large variation (Supplementary Fig. 1A). The mean doubling time of established iPSC clones was 29.67 ± 8.38 hours, which also did not show any correlation with age (Supplementary Fig. 1B). Male-derived iPSC lines had slightly longer doubling times (female: 29.29 ± 8.11 hours vs. male: 30.07 ± 8.64 hours; Fig. 2C). Interestingly, a few outliers with extremely long doubling times were observed, however, these were clones obtained from the same donors. The doubling times of the other clones from donors with such outliers were also longer than the average (Supplementary Fig. 1C). This is presumably due to a donor-derived characteristic; however, whether this was observed due to the genetic characteristics of the donor or the nature or condition of the source PBMCs remains unknown. Clones with extremely prolonged doubling times may require further evaluation, because of the possibility that genetic mutations or structural changes in the genome have occurred during the reprogramming process. In conclusion, we successfully established iPSCs from all recruited donors, and these iPSC lines were capable of proliferating in vitro, albeit with some variability.

### Evaluation of the basic properties of iPSCs

**Fig. 3:**
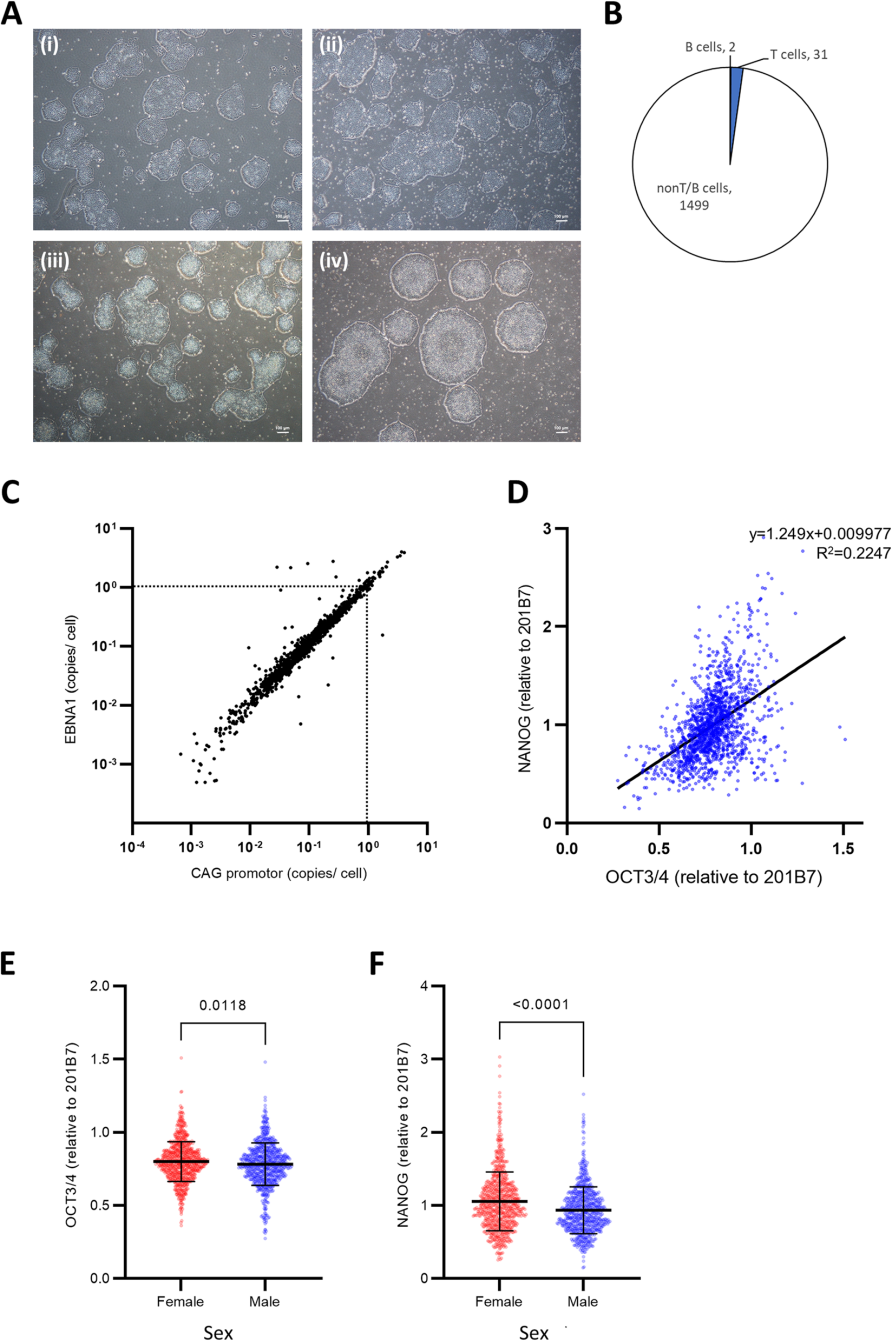
Basic characterization of established iPSCs. See also Supplementary Fig. 2. **A** Representative phase contrast images of iPSC colonies. iPSC clones from the patients with (i) Duchenne muscular dystrophy, (ii) Alexander disease, (iii) Dravet syndrome and (iv) Smith-Magenis syndrome are shown. Scale bars = 100 μm. **B **Estimation of the cell type from which iPSCs originated; *n* = 1,532. **C** Estimation of the number of residual copies of episomal vectors remaining in iPSCs, calculated based on the quantitative values of EBNA and CAG promotors. Dashed lines are drawn at the line corresponding to one copy of remaining vector per cell; *n* = 1,532. **D** Correlation between OCT3/4 and NANOG expression in each clone. The single regression equation is plotted on the graph; *n* = 1,532. **E**,** F** Comparison of OCT3/4 (**E**) and NANOG (**F**) expression by sex (female *n* = 798, male *n* = 734). Values for each clone have been plotted relative to the expression levels in control 201B7 iPSCs. Statistical analysis was performed using the unpaired t-test (**E**) and Mann–Whitney U test (**F**)

Next, we evaluated the basic properties of the established iPSCs. All iPSC clones had a morphology consistent with that of in *vitro* human pluripotent stem cells (representative images are shown in Fig. 3A). To determine the type of cells in the PBMCs from which each iPSC clone was derived, genomic recombination of the T cell receptor (TCR) and joining region of immunoglobulin heavy chain (JH) of iPSCs were evaluated. Interestingly, the majority (97.8%) of the iPSC clones had no recombination in both TCR and JH regions, indicating that they originated from non-T non-B hematopoietic cells (Fig. 3B).

The copy number of the episomal vectors remaining in the iPSC clones was also measured. Because all vectors used for reprogramming contained the CAG promoter and EBNA1 sequence, we evaluated the copy numbers of these two sequences per cell at passage 2. As a result, we observed that 96.3% of the clones had less than one copy of both CAG and EBNA promoters per cell (Fig. 3C). Thus, in most iPSC lines, the effect of plasmid persistence was deemed to be negligible.

### Expression of pluripotency-associated genes

We next assessed the expression of *OCT3/4* and *NANOG*, which are transcription factors expressed in undifferentiated pluripotent stem cells (Fig. 3D) [19, 20]. A weak correlation was observed between the expression levels of *OCT3/4* and *NANOG* in each iPSC line. No clones lacked the expression of OCT3/4 or *NANOG*, but a large variation in their expression levels was observed among the clones (relative to the average expression levels of *OCT3/4* and *NANOG* in control 201B7, wherein the expression levels of *OCT3/4* ranged between 0.27–1.51 (mean 0.79) and that of *NANOG* ranged between 0.15–3.03 (mean 1.00)). Interestingly, the expression levels of both *OCT3/4* and *NANOG* were significantly higher in female-derived clones (Fig. 3E and F). The association between age and the expression levels of these transcription factors was not evident (Supplementary Fig. 2A and B). Overall, all iPSC lines expressed the considerable amount of transcription factors specific to undifferentiated pluripotent stem cells.

### Database construction

Finally, to promote the use of our iPSC resource, we have built a database that provides access to the information pertaining to iPSCs produced in our resource projects, including this study (CiCLeD: CiRA iPS Cell Line Database). Brief information regarding the donors and iPSC clones described in this study can be freely accessed from CiCLeD constructed at the following URL (http://cicled.cira.kyoto-u.ac.jp/).

## Discussion

In this study, we established iPSCs from patients registered with designated intractable diseases, which is Japan's intractable disease support system, resulting in the establishment of a disease iPSC resource consisting of iPSCs established from patients with intractable diseases in a wide range of disease areas. The diseases from which iPSCs were established were diverse in terms of ICD-10 classification. The established iPSCs have been deposited in a public cell bank Riken Bioresource Center, and all researchers can potentially use these iPSCs for their own research pertaining to diseases.

Applying the medical history survey system of designated intractable diseases to the clinical information of iPSC donors will make it possible to obtain medical information in a uniform form for each disease and track donors in the future. In addition, if the patient database is accompanied by iPSC data, it would be possible to correlate disease phenotypes with the phenotypes at the cellular and genetic levels, thus providing very useful data for disease research. Unfortunately, medical information has been currently deposited in Japanese; therefore, it is necessary to make the dataset available globally in English.

It is already widely known that iPSCs are useful in rare disease research. As many rare diseases are hereditary, the establishment of iPSCs from the somatic cells of patients with rare genetic diseases can provide pluripotent stem cells that reflect the patient's genomic information. By differentiating these iPSCs into diseased cells, the pathology of each individual patient can be reproduced in vitro and analyzed in detail. Using patient-derived iPSCs, we can analyze the pathology of the rarest cases in details and reach a diagnosis. In fact, based on iPSC-derived phenotyping, it is possible to identify special genetic mutations that cannot be identified by whole-exon sequencing [21]. However, only about 300 designated intractable diseases, including rare intractable diseases, of the more than 7,000 rare diseases have been covered in the current study. In addition, not all disease-iPSCs included in designated intractable diseases have been established. Therefore, it is necessary to make continuous efforts to establish, evaluate, and deposit iPSCs for rare diseases, including designated intractable diseases.

Interestingly, there were sex-based differences in the expression levels of *NANOG*, an important transcription factor in PSCs. *SRY* gene on the Y chromosome is known to cause sex-dependent differences in global gene expression in human PSCs [22]. In addition, sex-based differences are known to cause differences in global demethylation during reprogramming [23]. Functional differences in differentiated cells derived from human PSCs have also been suggested previously [24]. These are small-scale studies, and our large cohort study suggests that sex-dependent differences can also affect those factors that are very important for the maintenance of pluripotency. The causes of this phenomenon and its consequences in terms of cell function remain to be elucidated.

The majority of iPSCs established from PBMCs lacked TCR and JH recombination, and were therefore considered to have been originated from non-T non-B cells. As the PBMC fraction also contains natural killer cells and monocytes in addition to T and B cells, these cells are thought to be the origin of iPSCs. This significant bias in the origin of iPSCs may be due to the fact that the cytokine setting used for the preculture of PBMCs did not include specific stimuli to proliferate T cells or B cells; for example, IL-2 anti-CD3 and anti-CD40 antibodies were not used. However, it was recently reported that preculture of PBMCs with similar cytokines expanded CD71-positive erythroblasts, and that these erythroblasts were the source of iPSCs [25]. Unfortunately, because we did not evaluate PBMCs prior to reprogramming, it is unclear as to which of these possibilities is more plausible.

In conclusion, we have established a large-scale iPSC panel of rare intractable diseases in Japan and deposited them in a public cell bank. These iPSCs are now widely available to the researchers and can prove to be a useful resource for research on rare intractable diseases. This study is a unique attempt to combine the basic resource of iPSCs for rare disease research with medical economic support measures for patients with intractable diseases. The future aim of such a system is to directly combine iPS cell-derived data with clinical findings and apply it to personalized medicine, including treatment and preventive medicine.

### Supplementary Information

**Additional file 1: Supplementary Table S1.** qRT-PCR primers used for iPSC characterization.

**Additional file 2: Supplementary Table S2.** Detailed donor information.

**Additional file 3: Supplementary Fig. 1.** Reprograming efficiency and doubling time of iPSCs, related to Fig. 2. **Supplementary Fig. 2.** Relationship of OCT3/4 and NANOG expression and donor age, related to Fig. 3.

## Data Availability

The datasets used and/or analyzed in this study are available in the CiCLED database. All iPSC clones can be distributed through the RIKEN BioResource Center to the extent consistent with Japanese law.

## Abbreviations

EU: European Union
US: The United States
iPSC: Induced pluripotent stem cell
ICD: International classification of diseases
TCR: T cell receptor
JH: Joining region of immunoglobulin heavy chain
NHO: National Hospital Organization
TRD: T cell receptor delta locus
IGH: Immunoglobulin heavy chain locus
