# Supplementary material for: A disease-specific iPS cell resource for studying rare and intractable diseases
Source: Inflamm Regen. 2023 Sep 8;43:43. doi: 10.1186/s41232-023-00294-2 (PMC10485998; doi:10.1186/s41232-023-00294-2)
Supplement: Supplementary file 3 — Additional file 3: Supplementary Fig. 1. Reprograming efficiency and doubling time of iPSCs, related to Fig. 2. Supplementary Fig. 2. Relationship of OCT3/4 and NANOG expression and donor age, related to Fig. 3. [file 41232_2023_294_MOESM3_ESM.docx]

**
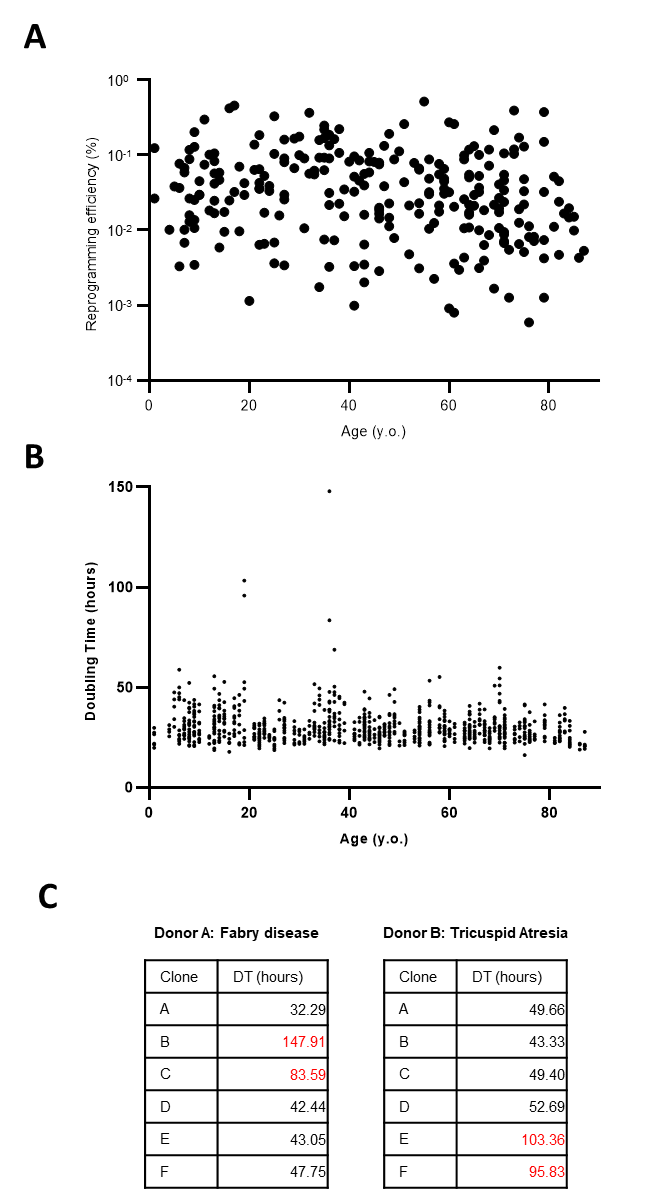
Supplementary Fig. 1. Reprograming efficiency and doubling time of iPSCs, related to Fig. 2.**

**(A)** Relationship between donor age and establishment efficiency; n=257. **(B)** Relationship between donor age and doubling time of iPSC lines. Note that multiple iPSC lines have been established from a single donor; n=1,039. **(C)** Doubling times for two donor-derived iPSC lines that showed particularly long doubling times as outliers in **Fig. 2C**.

**
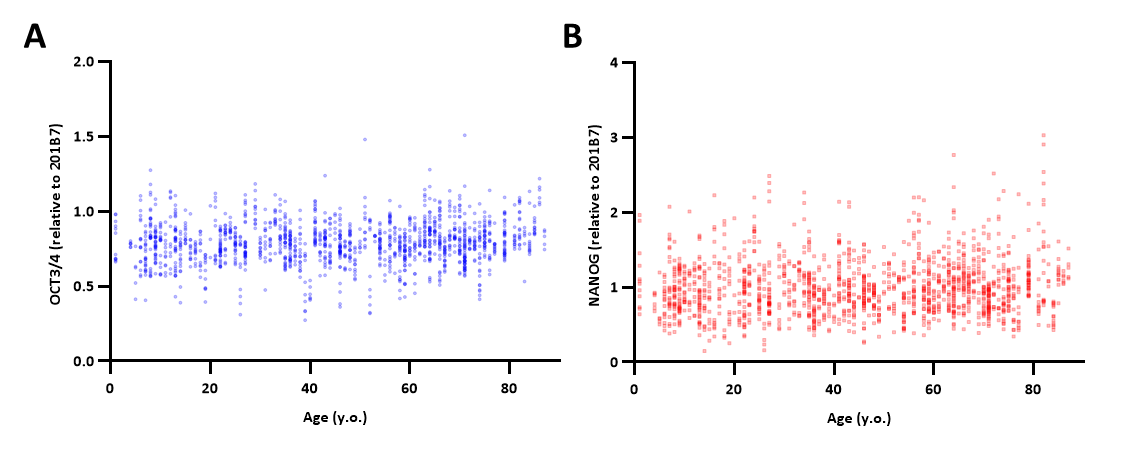
Supplementary Fig. 2. Relationship of OCT3/4 and NANOG expression and donor age, related to Fig. 3. (A,B)** Relationship between donor age and OCT3/4 (A) and NANOG (B) expression; n=1,532.
